# Cyanidin-3-O-Glucoside Alleviates Alcoholic Liver Injury via Modulating Gut Microbiota and Metabolites in Mice

**DOI:** 10.3390/nu16050694

**Published:** 2024-02-29

**Authors:** Lingfeng Zhu, Fuliang Cao, Zuomin Hu, Yaping Zhou, Tianyi Guo, Sisi Yan, Qiutao Xie, Xinxin Xia, Hongyan Yuan, Gaoyang Li, Feijun Luo, Qinlu Lin

**Affiliations:** 1 National Engineering Laboratory for Deep Process of Rice and Byproducts, Hunan Key Laboratory of Grain-Oil Deep Process and Quality Control, College of Food Science and Engineering, Central South University of Forestry and Technology, Changsha 410004, China; 2 Hunan Agricultural Product Processing Institute, Hunan Academy of Agricultural Sciences, Changsha 410125, China; 3 College of Forestry, Nanjing Forestry University, Nanjing 210037, China; 4 Hunan Academy of Agricultural Sciences, Changsha 410125, China

**Keywords:** cyanidin-3-O-glucoside, alcoholic liver injury, gut microbiota, metabolomics

## Abstract

Alcoholic liver disease (ALD) is primarily caused by long-term excessive alcohol consumption. Cyanidin-3-O-glucoside (C3G) is a widely occurring natural anthocyanin with multiple biological activities. This study aims to investigate the effects of C3G isolated from black rice on ALD and explore the potential mechanism. C57BL/6J mice (male) were fed with standard diet (CON) and Lieber-DeCarli liquid-fed (Eth) or supplemented with a 100 mg/kg/d C3G Diet (Eth-C3G), respectively. Our results showed that C3G could effectively ameliorate the pathological structure and liver function, and also inhibited the accumulation of liver lipids. C3G supplementation could partially alleviate the injury of intestinal barrier in the alcohol-induced mice. C3G supplementation could increase the abundance of *Norank_f_Muribaculaceae*, meanwhile, the abundances of *Bacteroides*, *Blautia*, *Collinsella*, *Escherichia-Shigella*, *Enterococcus*, *Prevotella*, *[Ruminococcus]_gnavus_group*, *Methylobacterium-Methylorubrum*, *Romboutsia*, *Streptococcus*, *Bilophila*, were decreased. Spearman’s correlation analysis showed that 12 distinct genera were correlated with blood lipid levels. Non-targeted metabolic analyses of cecal contents showed that C3G supplementation could affect the composition of intestinal metabolites, particularly bile acids. In conclusion, C3G can attenuate alcohol-induced liver injury by modulating the gut microbiota and metabolites, suggesting its potential as a functional food ingredient against alcoholic liver disease.

## 1. Introduction

Alcoholic liver disease (ALD) is a chronic disease caused liver damage by long-term excessive alcohol consumption. It refers to a broad spectrum of diseases, including fatty liver, alcoholic hepatitis, cirrhosis with their complications [1,2]. In the report of World Health Organization, 230 million people are current drinkers, and about 100 million of them are considered as heavy episodic drinkers [3]. This creates a substantial burden on global health. Previous studies show out that pathogenesis of ALD mainly includes oxidative stress, inflammation, and intestinal microbiota dysfunction and so on [4]. Currently, the available treatment approaches for ALD are limited, and there are usually side effects. Therefore, the exploration of novel therapeutic approaches to ALD has been of interest to researchers. Due to their wide range of anti-inflammatory and antioxidant activities, there has been concern about the use of natural active products in ALD treatment [5].

Black rice (*Oryza sativa* L. *Japonica*) is a nutritious food, also known as “medicine rice”. The black color of this rice is primarily attributed to the high concentration of anthocyanins, with cyanidin-3-O-glucoside (C3G) being the most prevalent, accounting for over 90.0% of the total anthocyanin content [6]. C3G from black rice has been approved pharmacological effects such as antioxidant, anti-inflammatory, anti-aging, anticancer [7,8,9,10]. Anthocyanin can bring a beneficial effect on human health, especially for the prevention of chronic and non-chronic diseases [11]. A previous study showed that anthocyanin-rich black rice extract could reduce lipopolysaccharide-induced inflammatory response in macrophage [12]. C3G also could alleviate non-alcoholic fatty liver disease by attenuating hepatic steatosis and activating NLRP3 inflammasome [13]. Oral administration of C3G could improve glucose tolerance and reduced lipid accumulation in the high-fat diet mice [14]. It is not clear whether C3G has protective effect on ALD.

Some investigations show that the changes of microbial community structure and quantity play an important role in the development of ALD. The abundance of *Bacteroidetes* in the alcohol-fed mice were significantly increased and *Firmicutes* were decreased [15]. Furthermore, the gut microbiota can interact with the liver via the gut-liver axis, for example, lipopolysaccharide, a component of gut Gram-negative bacteria, enters the liver via the portal vein to promote inflammation, eventually promote the development of ALD [16]. Black rice supplementation could restore microbial richness in the intestine, which is related to improved lipid metabolism and obesity [17]. We speculate that the function of black rice may be related to its main substance, C3G. Therefore, the aim of this study was to investigate the effect of C3G on alcoholic liver injury in mice and to explore its possible mechanism through gut microbiota.

## 2. Materials and Methods

### 2.1. Animal and Experimental Design

**Figure 1:**
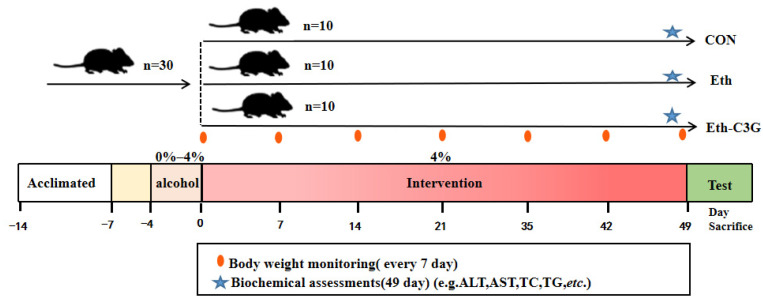
Experimental protocol for the effect of C3G on ALD mice. C57BL/6J mice were randomly divided into three groups (*n* = 10): Control (CON), alcohol (Eth), and alcohol with C3G (Eth-C3G) groups after adaptation period for 7 days. Then the all mice were fed with a liquid diet for 3 days, next the Eth and Eth-C3G group mice were fed alcohol liquid diet (0% to 4%, gradually) adaptively for another 4 days, then maintained at 4% (*v*/*v*) levels for the next 7 weeks, the Eth-C3G group mice were supplemented with C3G (100 mg/kg/day) until the end of the experiments.

C57BL/6J male mice (8-week-old) were obtained from Hunan SJA Laboratory Animal Co., Ltd. (SLAC, Changsha, China). All mice were accommodated to a environment at 24–25 °C of temperature, 40–60% of humidity and 12 h dark/light cycle. The animal experimental procedures are accorded to the Animal Care and Use Guidelines, the use and ethics of experimental animals were approved by Hunan Prima Pharmaceutical Research Center (HNSE2021[5]068, Liuyang, Changsha, China). After 1 week of acclimatization, 30 mice of similar weight were randomly divided into three groups (each group has 10 mice): control group (CON), ethanol group (Eth), and ethanol + C3G (100 mg/kg/day; C3G came from black rice, 93.0% purity; YiRui Biotech Company, Chengdu, China) group (Eth-C3G), The alcohol-induced liver injury mouse model was followed the method of Guo et al. [18] with a little modification. Mice in the Eth and Eth-C3G groups were acclimated to an ethanol diet for 4 days, in which ethanol was gradually added to the liquid diet (0% to 4%), then the ethanol concentration was maintained at a 4% (*v*/*v*) for the subsequent 7 weeks (Figure 1). Mice were euthanized with ethyl ether and blood, tissue samples and cecal content were collected. Tissue and cecal content samples were stored in liquid nitrogen.

### 2.2. Biochemical Assays

The blood samples of mice were centrifuged at 3000 rpm/min for 15 min and the serum was obtained. Total cholesterol (TC), total triglyceride (TG), low-density lipoprotein cholesterol (LDL-c), and high-density lipoprotein cholesterol (HDL-c) contents in serum samples were detected by Rayto Biochemical Analyzer Chemray 240 (Shenzhen, China) according to kit instructions. The malondialdehyde (MDA) content and enzyme activities of aspartate aminotransferase (AST), alanine aminotransferase (ALT), superoxide dismutase (SOD) and glutathione peroxidase (GSH-Px) were measured by Microplate reader (Epoch, BioTeK, Winooski, VT, USA) according to the instruction of kit (Nanjing jiancheng bioengineering Institute, Nangjing, Jiangsu, China).

### 2.3. Histological Evaluation

The liver and colon tissues were separated and fixed in a 4% (*v*/*v*) solution of paraformaldehyde for 24 h. Then, the samples were dehydrated with ethanol and paraffin-embedded. Finally, paraffin sections were stained with hematoxylin and eosin (H&E). In order to visualize liver fat deposition more clearly, frozen sections of liver tissue samples were stained with Oil Red O. Images were captured on a microscope imaging system (Nikon, Tokyo, Japan).

### 2.4. Immunofluorescence Assessment

Fixed colon tissues were paraffin embedded and sectioned (5 μm thickness). The paraffin sections were dewaxed and the tissue sections were treated with an EDTA antigen repair buffer, followed by BSA treatment for 30 min. After removing the blocking solution, the primary antibody (anti-ZO-1 or anti-Claudin-1) was added to the slides, and incubated at 4 °C overnight. Then PBS was used to wash unbinding primary antibody. Secondary antibody (HRP-conjugated goat anti-rabbit immunoglobulin G) was added into the slides at room temperature for 1 h. Then, the corresponding BSA was added into the slides for 10 min in darkroom. Finally, DAPI was added into the slides for 12 min in darkroom, then autofluorescence quencher were added into the slices. After PBS decolorization, images were acquired by inverted fluorescence microscopy (Olympus, Tokyo, Japan).

### 2.5. Gut Microbiota Analysis

16S rRNA sequencing is commonly used to analyze changes in the gut microbiota. The aim of this study was to investigate the effects of different treatments on bacteria in cecal contents. Following the manufacturer’s instructions, the total DNA of cecal microbiota were extracted from the CON, Eth, and Eth-C3G groups by a MagPure DNA LQ Kit (Magen, Foshan, Guangdong, China). 16S rRNA Sequencing was performed in Shanghai Ouyi Biomedical Technol Co., Ltd., Shanghai, China. The results were analyzed at OE Biotech Cloud Platform (https://cloud.oebiotech.com/task/) (accessed on 5 May 2022).

### 2.6. Metabolomic Analysis of Cecal Contents

All samples were measured using the Agilent Technologies 7890B Gas chromatography system coupled with the Agilent Technologies 5977A MSD system (Agilent Technologies, Santa Clara, CA, USA). 60 mg cecal content sample was added to Eppendorf tube. Samples were homogenized with cold methanol (360 μL) and internal standard (40 μL, 0.3 mg/mL 2-chlorophenylalanine) and were sonicated for 30 min. After the samples were rotated for 2 min and sonicated with 300 μL chloroform and 600 μL water for 30 min, and the mixture was centrifuged at 13,000 rpm for 10 min at 4 °C. Finally, the supernatant was dried under vacuum, mixed with 80 μL of methoxyamine, and oximated for 90 min in the dry supernatant; then 80 μL BSTFA and 20 μL n-hexane solution were added for 60 min at 70 °C. Metabonomics analysis was performed by gas chromatography-mass spectrometry (GC-MS).

The peak recognition, peak alignment, waveform filtering and missing interpolation of the original data were analyzed by using MS-DIAL software and metabolite modeling was based on the Lug database. The data matrix sample information, the peak name of each substance, the retention time, the mass-to-charge ratio and the signal strength were acquired. Results were analyzed using the OE biotechnology cloud platform (https://cloud.oebiotech.com/task/) (accessed on 22 January 2023).

### 2.7. Statistical Analysis

The data were expressed as means ± SD, and all data in this study were analyzed by SPSS software (version 25.0, Chicago, IL, USA). One-way ANOVA and Tukey’s multiple range test were used to analyze the differences among the groups. Tamhane’s T2 test was used for post hoc multiple comparisons when variance was unequal (statistical significance, *p* < 0.05). Our bioinformatics analysis including species classification, richness and diversity analysis was performed on the OE biotechnology cloud platform, a data analysis platform. The correlations between microbiota and phenotype or metabolites were analyzed using Spearman’s correlation analysis.

## 3. Results

### 3.1. Effects of C3G Administration on Body Weight and Liver Index

**Figure 2:**
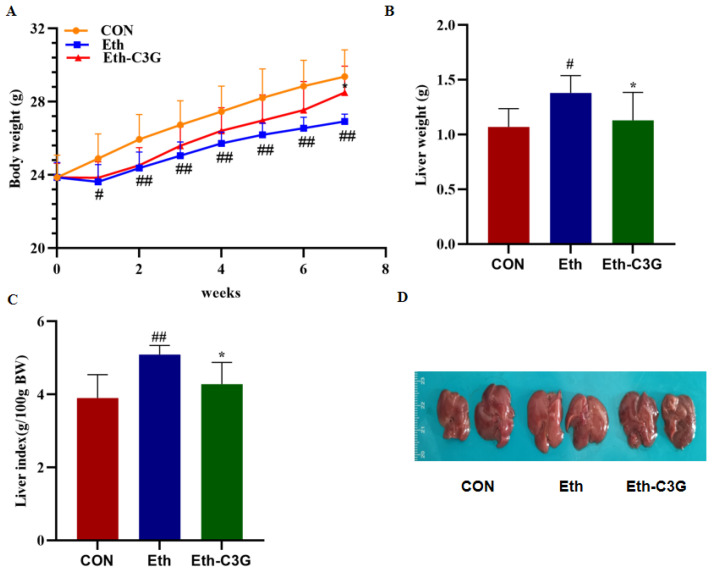
Effects of C3G on macroscopic phenotype of alcoholic liver injury in mice. (**A**) The change of body weight during 7 weeks; (**B**) liver weight; (**C**) liver index (liver weight: body weight × 100); (**D**) images of representative liver in each group. CON: control group; Eth: alcohol model group; Eth-C3G: alcohol and C3G treatment group. The values represent mean ± SD, #: *p* < 0.05, ##: *p* < 0.01, compared with the CON group; *: *p* < 0.05, compared with the Eth group.

The changes of mice body weight during the experimental period are shown in Figure 2A. The average body weight of the mice in Eth group was significantly lower than the CON group during the first week (*p* < 0.05), and this trend was maintained throughout the experiment. Compared to the Eth group, C3G supplementation could increase body weight gain of mice from 26.92 ± 0.38 g of Eth group to 28.51 ± 1.37 g of Eth-C3G group at the end of the 7th week. Liver weight and liver index were measured in the three groups (Figure 2B,C). Compared to the CON group, the Eth group exhibited a significant increase in liver weight and liver index (*p* < 0.05). However, this abnormality was mitigated by the administration of C3G treatment. The color of livers was cream in Eth group mice. Compared with Eth group, C3G treatment obviously reduced the size of livers and recovered the liver color similar to CON group (Figure 2D). The findings demonstrate that C3G can reduce the damage of alcohol to the body and liver enlargement in the mice of Eth group.

### 3.2. Effects of C3G on the Hepatic Histopathological Changes

**Figure 3:**
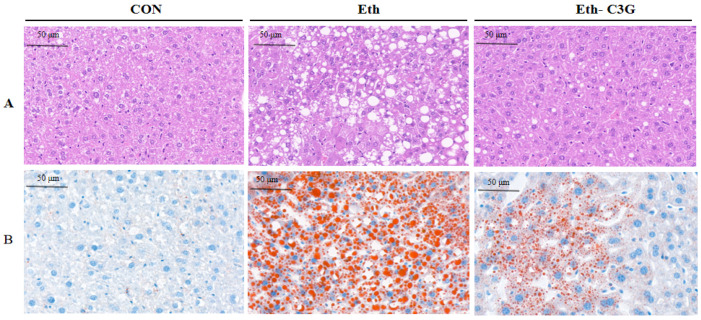
Pathological changes in liver tissue. (**A**) hematoxylin-eosin staining; (**B**) liver stained with Oil Red O; CON: control group; Eth: alcohol model group; Eth-C3G: alcohol and C3G treatment group.

The pictures of H&E staining and Oil Red O staining intuitively showed the damage of alcoholic liver (Figure 3A,B). The hepatocytes irregular arrangement and extensive macrovesicular lipid droplets were observed in Eth group mice. After C3G intervention for 7 weeks, these morphological and pathological symptoms were visibly alleviated. The results showed C3G treatment reduced lipid deposition in hepatocytes and restored the cell morphology.

### 3.3. Effects of C3G on Biochemical Metabolic-Related Parameters

**Figure 4:**
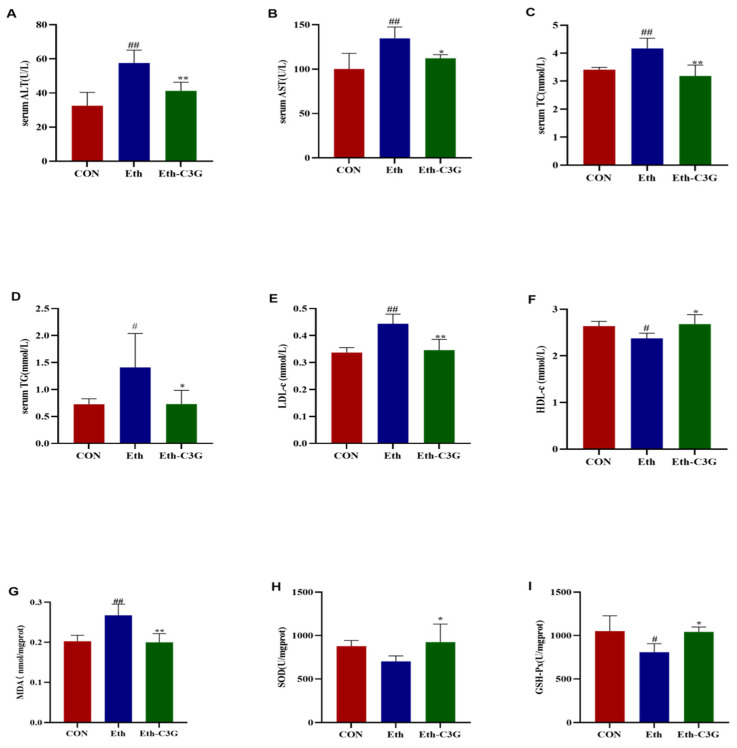
Effects of C3G on serum levels of liver enzymes, liver lipid profile and antioxidants levels of liver in the ALD mice. Enzymatic activities of ALT (**A**) and AST (**B**) in serum; concentration of total cholesterol (**C**) and triglycerides (**D**); serum low density lipoprotein cholesterol (LDH-c) (**E**); serum high density lipoprotein cholesterol (HDH-c) (**F**); MDA in liver (**G**); SOD in liver (**H**); GSH-Px in liver (**I**). The values represent mean ± SD. CON: control group; Eth: alcohol model group; Eth-C3G: alcohol and C3G treatment group. #: *p* < 0.05, ##: *p* < 0.01, compared with the CON group; *: *p* < 0.05, **: *p* < 0.01, compared with the Eth group.

Alcohol treatment had significant effects on liver-specific enzymes. The enzyme activities of ALT and AST in the Eth group mice were 1.77 and 1.34 folds higher than these indexes in CON group mice, respectively (Figure 4A,B), indicating the damage of liver cell. However, these enzymes activities of Eth-C3G group mice were significantly lower than those of the Eth group mice (*p* < 0.01 and *p* < 0.05, respectively). It suggests that C3G has a potential protective effect on alcoholic liver injury.

After the mice were fed with alcohol for 7 weeks, the TG, TC, and LDL-c concentration in serum significantly increased compared to the CON group (higher 94.35%, 22.14%, and 31.77%, respectively) (Figure 4C–E). In this study, compared with Eth group, C3G significantly decreased serum TG, TC, and LDL-c levels by 48.20%, 23.61%, and 21.99%, respectively. The level of HDL-c in the Eth group significantly decreased compared with that in the CON group (*p* < 0.05) (Figure 4F) and C3G supplementation reversed this trend (*p* < 0.05). These data were consistent with the result of Oil Red O staining in liver tissues, suggesting that the model of alcohol-induced liver injury was established successfully in this study. These results indicate that the consumption of C3G effectively alleviate alcohol-induced dyslipidemia in mice.

MDA is alcohol-induced by-product of lipid peroxidation, and its concentration in the liver indirectly indicates the extent of liver injury. The liver MDA concentration was significantly higher in the Eth group (0.267 ± 0.022 nmol/mg protein) compared to the CON group (0.202 ± 0.015 nmol/mg protein) (*p* < 0.01). However, the liver MDA concentration was suppressed (0.202 ± 0.020 nmol/mg protein) (*p* < 0.01) after C3G treatment (Figure 4G). SOD and GSH-Px in hepatocytes are crucial in protecting against alcohol-induced liver damage, as they have a strong capacity to scavenge free radicals generated within the body. When the free radical content in the organism exceeds the limit of the oxidative barrier defense, the organism will be subjected to oxidative damage [19]. In this study, excessive alcohol consumption suppressed the activities of hepatic SOD and GSH-Px. Consumption of C3G obviously elevated the hepatic activities of those antioxidant enzymes (Figure 4H,I). It suggests that C3G has the potential to prevent alcohol-induced liver damage by reducing the oxidative stress level.

### 3.4. Effects of C3G on Intestinal Histopathological Feature

**Figure 5:**
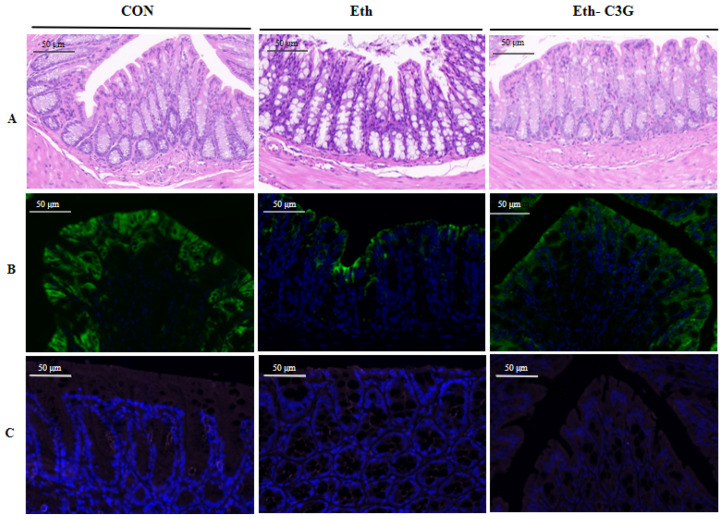
Effects of C3G on intestinal barrier damage in ALD mice. (**A**) Histopathological analysis of colon stained with H&E (20× magnification); (**B**,**C**) immunofluorescence staining of tight junction claudin-1 (green) and ZO-1 (pink) in the colon (20× magnification). CON: control group; Eth: alcohol model group; Eth-C3G: alcohol and C3G treatment group.

Histological analysis indicated that alcohol treatment damage intestinal epithelial villi in the Eth group mice. The surrounding villi became thinning and atrophy, and the gross morphology was abnormal as well. These pathological and morphological changes were alleviated by C3G treatment to some extent (Figure 5A). The expressions of Claudin-1 and ZO-1 in colon were analyzed by immunofluorescence staining (Figure 5B,C). The expressions of Claudin-1 and ZO-1 in the Eth group were decreased compared with the CON group, suggesting that the intestinal barrier is damaged. After C3G intervention for 7 weeks, the distributions of Claudin-1 and ZO-1 were visibly restored.

### 3.5. Effects of C3G on Gut Microbiota in Mice

**Figure 6:**
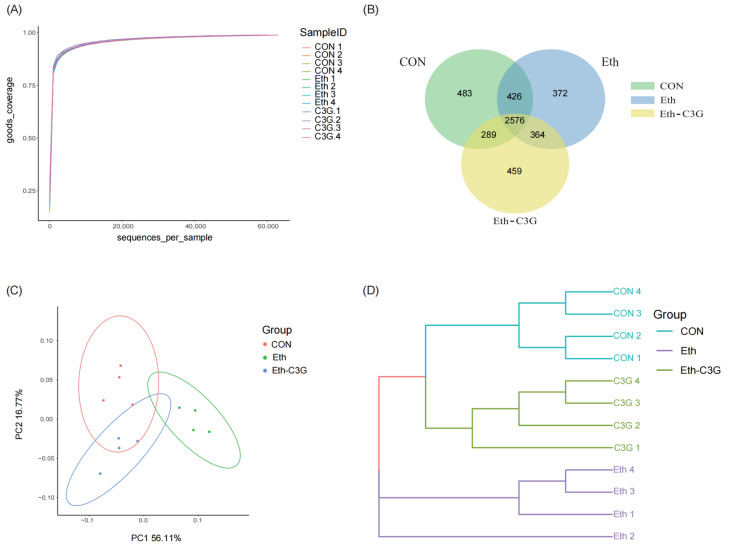
(**A**) OTU Dilution curve analysis; (**B**) Venn analysis; (**C**) PCoA; (**D**) hierarchical clustering tree on the OTU level by weighted_unifrac. CON: control group; Eth: alcohol model group; Eth-C3G: alcohol and C3G treatment group.

The gut microbiota is tightly related to host physiology and immunity [20]. Various environmental factors including diet, disease and medication, exert an influence on the composition of gut microbiota [21,22]. Among these factors, diet significantly contributes to the modification of gut microbiota [23]. To evaluate the impact of C3G intervention on the change of gut microbiota, 16S rRNA sequencing was used to analyze the cecal contents of mice. Alpha diversity analysis of goods_courage curves for each sample reached the saturation plateau (Figure 6A). This suggested that our subsequent experiments had a high degree of confidence. As presented by the Venn diagram in Figure 6B, a total of 4969 operational taxonomic units (OTUs) at 97% similarity were obtained. 2576 OTUs overlapped in 4969 OTUs in all of the groups, and there were 372 and 459 distinct bacteria in the Eth and Eth-C3G groups, respectively. Distinct clustering of the microbiota compositions of the three group were observed using the principal co-ordinates analysis (PCoA) (Figure 6C). This result was also presented in Hierarchical clustering analyses (Figure 6D). These findings collectively suggested that the disordered colony structure of Eth-treated mice was restored by C3G treatment, making it more similar to that of the CON group mice.

**Figure 7:**
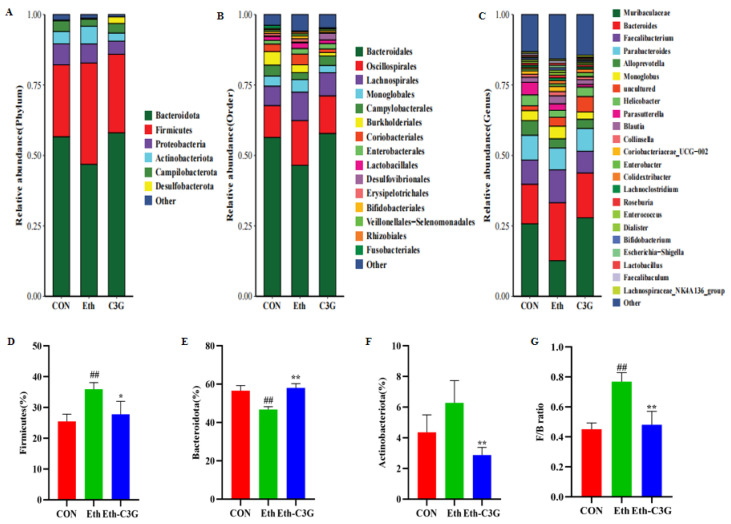
(**A**) Percent of community abundance at the phylum level; (**B**) percent of community abundance at the order level; (**C**) percent of community abundance at the genus level; (**D**) *Firmicutes* abundance (**E**) *Bacteroidota* abundance; (**F**) *Actinobacteroidota* abundance; (**G**) *Firmicutes/Bacteroidota* (F/B) ratio. CON: control group; Eth: alcohol model group; Eth-C3G: alcohol and C3G treatment group. The values represent mean ± SD, *n* = 4. ##: *p* < 0.01, compared with the CON group; *: *p* < 0.05, **: *p* < 0.01, compared with the Eth group.

To assess the overall composition and structure of gut microbiota among different groups, and specifically how they respond to C3G supplementation, we analyzed the distribution and abundance of bacterial taxa at each taxonomic level. At the phylum level, *Firmicutes* and *Bacteroidota* were the dominant phyla, accounting for more than 82% of the total microflora (Figure 7A). Alcohol stimulation significantly increased the ratio of *Firmicutes* to *Bacteroidota* compared with the CON group (Figure 7D,E). C3G treatment effectively downregulated *Firmicutes* relative abundance while upregulated *Bacteroidota* relative abundance. Ethanol treatment increased the relative abundance of *Actinobacteriota*. C3G treatment protected against this trend (*p* < 0.01) (Figure 7F). The *Firmicutes/Bacteroidota* (F/B) ratio was considered to be an important indicator for metabolic disorder [24]. The Eth groups had a larger F/B ratio than the CON and Eth-C3G groups (*p* < 0.01) (Figure 7G).

**Figure 8:**
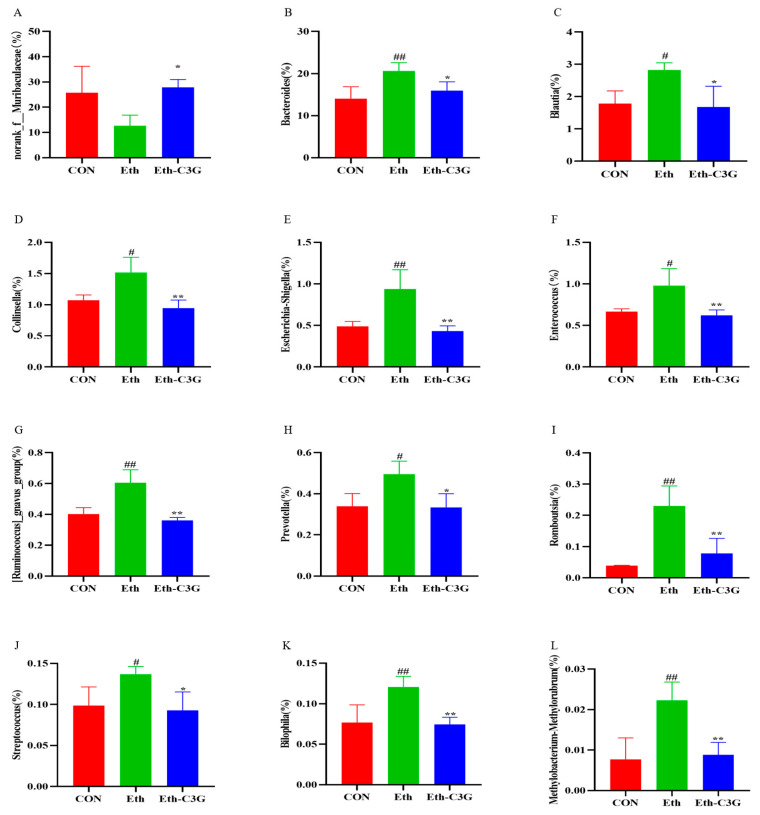
Representative genus the relative abundance (%) of (**A**) Norank_f_Muribaculaceae; (**B**) Bacteroides; (**C**) Blautia; (**D**) Collinsella; (**E**) Escherichia-Shigella; (**F**) Enterococcus; (**G**) [Ruminococcus]_gnavus_group; (**H**) Prevotella; (**I**) Romboutsia; (**J**) Streptococcus; (**K**) Bilophila; (**L**) Methylobacterium-Methylorubrum. CON: control group; Eth: alcohol model group; Eth-C3G: alcohol and C3G treatment group. The values represent mean ± SD, *n* = 4. #: *p* < 0.05, ##: *p* < 0.01, compared with the CON group; *: *p* < 0.05, **: *p* < 0.01, compared with the Eth group.

C3G also demonstrated promising effects in reversing some of alcohol-induced changes in the composition of gut microbiota, including *Bacteroidales* (*p* < 0.01), and *Lactobacillales* (*p* < 0.01) at the order level (Figure 7B). At the genus level (Figure 7C), *Muribaculaceae*, *Bacteroides*, *Faecalibacterium*, *Parabacteroides*, and *Alloprevotella* were enriched. C3G treatment inverted the downregulated abundance of *Norank_f_Muribaculaceae* stimulated by alcohol (*p* < 0.05) (Figure 8A). Conversely, C3G treatment decreased the relative abundances of *Bacteroides* (*p* < 0.05), *Blautia* (*p* < 0.05), *Collinsella* (*p* < 0.01), *Escherichia-Shigella* (*p* < 0.01), *[Ruminococcus]_gnavus_group* (*p* < 0.01), *Enterococcus* (*p* < 0.01), *Prevotella* (*p* < 0.05), *Romboutsia* (*p* < 0.01), *Streptococcus* (*p* < 0.05), *Bilophila* (*p* < 0.01), and *Methylobacterium-Methylorubrum* (*p* < 0.01) (Figure 8B–L), which were significantly enhanced in the Eth group. The above results indicated that C3G has the potential to improve intestinal microbial dysbiosis occurred in chronic alcohol-exposed mice.

### 3.6. Effects of C3G on Profiles of Metabolites in Cecal Contents

**Figure 9:**
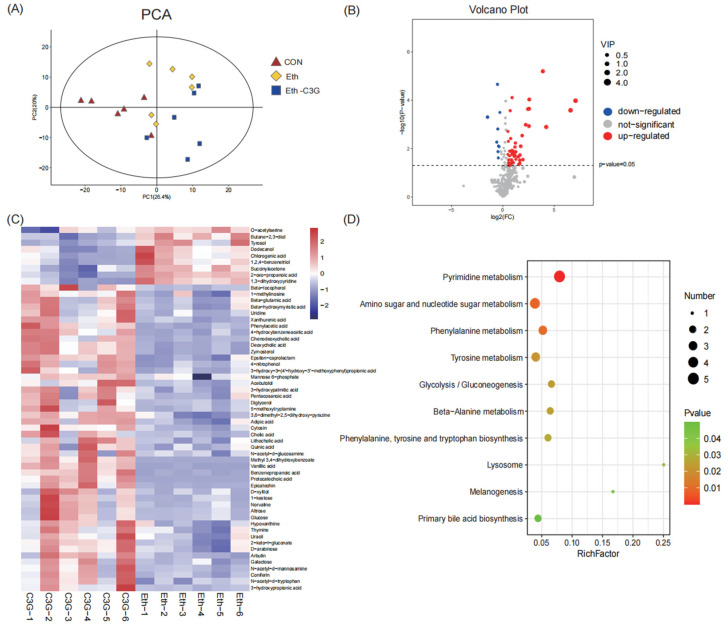
Cecal contents metabolomic profiling by non-targeted metabolism method based on GC-MS. (**A**) PCA score plot for the CON, Eth and Eth-C3G groups; (**B**) volcano score plot for the Eth and Eth-C3G groups; (**C**) heatmap of the concentration of significantly different metabolites (VIP > 1.0, *p* < 0.05) between Eth and Eth-C3G groups; (**D**) metabolic pathway impact prediction based on Kyoto Encyclopedia of Genes and Genomes (KEGG). CON: control group; Eth: alcohol model group; Eth-C3G: alcohol and C3G treatment group; *n* = 6.

Metabolomics can provide quantitative information on full changes in the metabolic function [25]. Cecal contents were analyzed using GC-MS to find the different metabolites, further to explore the key metabolites of C3G intervention against alcoholic liver injury. Principal component analysis (PCA) plots showed clear separation between the Eth group and other groups, which means the gut microbiota in different groups have obvious changes (Figure 9A). According to VIP value > 1.0 and *p* value < 0.05, a total of 57 metabolites were successfully identified in the cecal contents between the Eth and Eth-C3G groups (Figure 9B). Compared with the Eth group, 48 metabolites were significantly upregulated and 9 metabolites were significantly downregulated in Eth-C3G group (Figure 9C). In this study, we found C3G treatment significantly elevated the proportion of bile acids, such as deoxycholic acid (DCA), chenodeoxycholic acid (CDCA), lithocholic acid (LCA), and cholic acid (CA). The potential biomarkers were used for pathway analysis using MetaboAnalyst 4.0 and Kyoto Encyclopedia of Genes and Genomes (KEGG). Ten metabolic pathways (rich factor > 0, *p* < 0.05) were enriched between the Eth and the Eth-C3G groups, including pyrimidine metabolism (rich factor = 0.0769), amino sugar and nucleotide sugar metabolism (rich factor = 0.0370), phenylalanine metabolism (rich factor = 0.05), tyrosine metabolism (rich factor = 0.0385), glycolysis/gluconeogenesis (rich factor = 0.0645), beta-alanine metabolism (rich factor = 0.0625), phenylalanine (rich factor = 0.0588), lysosome (rich factor = 0.25), melanogenesis (rich factor = 0.1667), and primary bile acid biosynthesis (rich factor = 0.0426) (Figure 9D).

### 3.7. Correlation Analysis

**Figure 10:**
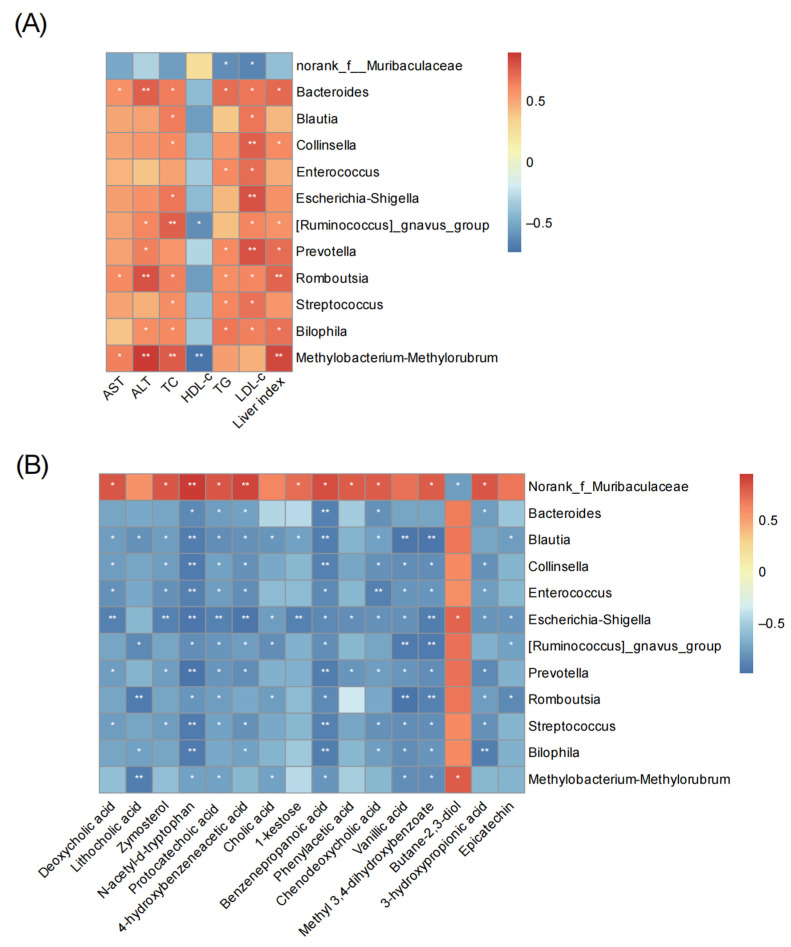
Spearman correlation (un-adjusted *p* value). (**A**) Between the genus and biochemical variables; (**B**) between the genus and cecal metabolites. Heatmap shows the value of the correlation coefficient. * indicates a significance of the associations (*: *p* < 0.05, **: *p* < 0.01, respectively). Red squares indicate positive correlation and blue squares indicate negative correlation.

Alterations of gut microbiota and their metabolites are the main cause of host metabolic disturbances [26]. Therefore, the correlation heatmap was established to identify the potential association between gut microbiota disturbances and changes in cecum metabolites. Correlation analyses was performed with both the genus vs. biochemical variables and the genus vs. cecal metabolites (Figure 10A,B, respectively). The 12 kinds of gut microbiota were reported to be related to obesity, inflammation, hyperlipidemia, and bile acid metabolism. Our correlation results indicated that the relative abundance of *Norank_f_Muribaculaceae* exhibited a negative correlation with serum lipid levels, while 11 other genera showed a positive correlation with liver index and serum lipid levels, particularly TC and LDL-c levels. These correlation analyses implied that the variations in bacterial abundance were closely associated with lipid metabolism (Figure 10A).

As showed relationship between gut microbiota and cecal typical metabolites (Figure 10B), the genera decreased after C3G intervention such as *Bacteroides*, *Blautia*, *Collinsella*, *Escherichia-Shigella*, *Enterococcus*, *[Ruminococcus]_gnavus_group*, *Prevotella*, *Romboutsia*, *Streptococcus*, *Bilophila*, and *Methylobacterium-Methylorubrum*. They were negatively correlated with LCA, DCA, CDCA, CA and other metabolites of C3G in vivo (e.g., protocatechoic acid, vanillic acid). But the *Norank_f_Muribaculaceae* showed the opposite trend. This correlation analyses illustrated that C3G might inhibit bacterium that are not conducive to liver repair by its metabolites and increasing bile acids.

## 4. Discussion

ALD is one of the major diseases of harm to human health. This study utilized a combined approach of microbiome analysis and metabolomics to explore the protective effects of C3G against alcoholic liver injury and elucidate its possible mechanism.

In the current study, we found C3G supplementation significantly attenuated alcohol-induced liver lipid and serum lipid accumulation. Similar to most studies, alcohol intake increased liver index and the contents of serum TG, TC, and LDL-c [27,28]. After C3G consumption, liver index, serum TG, TC, and LDL-c concentrations of the alcohol-exposed mice were significantly decreased, and significantly increased HDL-c. The results of H&E staining and Oil Red O staining clearly demonstrated that C3G effectively reduced hepatic lipid accumulation. This illustrated that C3G intervention had a modulatory effect on lipid metabolism, Lyu et al. also discovered casein-C3G could prevent the accumulation of liver lipid in HFD-fed mice [29].

Long-term drinking not only produced reactive oxygen, but also weakened the antioxidant system. Our results confirmed that the intervention of C3G increased the activities of SOD and GSH-Px in liver, and decreased the content of MDA, which suggested that C3G could enhance antioxidant system in liver and reduce the membrane lipid peroxidation. This was related to the strong antioxidant activity of C3G. Ye et al. reported that C3G alleviated oxidative stress in islets by the improve activities of SOD and catalase [30].

Disruption of the intestinal barrier is considered a significant damage of alcohol abuse [16]. Damaged intestinal barrier aggravated liver damage by passing intestinal endotoxin through the intestinal-hepatic axis [31]. Our study demonstrated that the intervention of C3G restored the alcohol-induced intestinal barrier damage by increasing the expressions of Claudin-1 and ZO-1 proteins. C3G and its metabolites were found to have a protective effect on the intestinal barrier in vitro [32,33]. Some researchers proposed that the disruption of intestinal barrier might be attributed to the alcohol metabolites generated by gut microbiota or the dysbiosis of gut microbiota [34,35]. Therefore, relevant researches were carried out in this study.

Alcohol has been reported to cause changes in the gut microbiota, which in turn lead to an imbalance in pathogenic and symbiotic organisms in the microbiota, thereby damaging liver through the enterohepatic axis [16,36]. There were also studies showing that dietary interventions can regulate intestinal microbiota [37,38]. In our study, gut microbiota played a huge role for C3G to ameliorate alcohol-induced liver injury. An increase in the F/B ratio is a feature of the intestinal microbiota of the ALD mouse model [39,40]. In our research, alcohol significantly increased the proportion of *Firmicutes* and decreased the proportion of *Bacteroidetes*, and the intervention of C3G reversed the trend. In diet-induced insulin resistant rats, C3G demonstrated similar effects on gut microbiota [41]. As the largest genus in mouse feces, the abundance of *Norank_f_Muribaculaceae* was reduced in the mice with alcohol-induced metabolic disorders or in the mice with colitis [42,43,44]. In the present study, C3G intervention significantly increased the abundances of *Norank_f_Muribaculaceae,* which was involved in the formation of the mucus layer, and ameliorated the gut barrier function [45]. Meanwhile, alcohol induced significant increase of some bacteria, such as *Bacteroides*, *Blautia*, *Collinsella*, *Escherichia-Shigella*, *Methylobacterium-Methylorubrum Enterococcus*, *[Ruminococcus]_gnavus_group*, *Prevotella*, *Romboutsia*, *Streptococcus and Bilophila,* they were reported to promote the liver damage. C3G supplementation could suppress their growth. Meanwhile these bacteria were positively correlated with serum lipids. *Bacteroides* is highly correlated with enteritis, and its increase was considered as an indicator to identify patients with alcohol use disorder [46,47]. Some recent studies have shown that the abundance of *Blautia* was increased in patients with obesity or ALD [48,49,50]. Furthermore, *Blautia* has a positive correlation with serum TG accumulation in our study, suggesting its possible participation in the lipid accumulation. *Collinsella* was reported to be highly abundant in obese mice [51,52]. *Escherichia-Shigella* and *Enterococcus* are usually considered to be pro-inflammatory bacteria, and *Escherichia-Shigella* was participated in obesity-associated metabolic dysfunction [37,53]. *Enterococcus* was reported to increase the mortality in patients with alcoholic hepatitis, it secreted cytolysin-a two-subunit exotoxin, thus leading to the hepatocyte death and liver injury [54]. *[Ruminococcus]_gnavus_group* was linked with bile acid metabolism [55]. Compared with healthy controls, the abundances of *Blautia*, *Prevotella*, and *Ruminococcus* were elevated in the children with non-alcoholic fatty liver disease and non-alcoholic steatohepatitis [56]. Berberrubine treatment decreased *Romboutsia* abundance, a obesity-related bacteria [57]. *Streptococcus* might promote the production of AST, which could be used as a marker to evaluate liver injury [58]. Yeast β-glucan had a significant impact on reducing the abundance of *Bilophila* in human cohorts, a microorganism associated with obesity. *Methylobacterium-Methylorubrum* was significantly enriched in post-hepatectomy liver failure [59]. The interactions among bile acid composition, gut microbiota, and metabolic phenotype were confirmed in the healthy and high-fat diet mice [60]. Collectively, C3G may exert a protective role against alcohol-induced liver injury by modulating the balance of the gut microbiota.

When the gut microbiota is subjected to external influences that produce changes in abundance, this may lead to changes in host metabolites as well. At the same time, the compounds and their metabolites also affect the gut microbiota. In recent years, more and more investigations paid attention to metabolites including bile acids [61]. In the present study, we focused on the metabolites that were affected by C3G in alcohol-exposed mice. Possibly due to the two hydroxyls on the B ring, C3G has strong antioxidant activity [62]. Previous studies proposed that C3G bioactivities primarily derived from its metabolites [63]. More than 20 kinds of C3G metabolites (C3G-Ms) in serum have been identified [64]. Among them, protocatechuic acid [65,66,67], vanillic acid [68,69] or their derivatives are the main bioactive metabolites, which have antioxidant and anti-inflammatory activities. The metabolites of C3G detected in this study were protocatechoic acid, methyl 3,4-dihydroxybenzoate, vanillic acid, 4-hydroxybenzeneacetic acid, et al., which may increase C3G bioavailability and contribute to the repair of gut mucosal barrier and intestinal microbiota disorder [70,71]. Protocatechoic acid reduced the abundance of *Ruminococcus* and *Prevotella* in lipopolysaccharide-challenged piglets [70]. *Lycium barbarum* extract (rich in protocatechoic acid) decreased the abundance of *Ruminococcus* and *Blautia* in diabetic rats [72]. Protocatechoic acid decreased the abundance of *Enterococcus*, which had a positive correlation with serum AST and ALT [66]. The intestinal microbiota participated in metabolism or synthesis of bile acids, meanwhile, bile acids impacted the structure and function of bacterial community, and participated in the lipid metabolism [61]. In this study, C3G increased the concentrations of CA, CDCA, DCA, and LCA in cecal contents. The concentrations of DCA and LCA in cecal contents were decreased in alcohol-associated hepatitis [73]. Correlation analysis showed that 11 genera were negatively correlated with bile acids levels, they were not conductive to the recovery of liver injury. The previous studies found that the increased level of ileal conjugated bile acids (BAs) reduced hepatic cholesterol and lipogenesis [38]. Thus, C3G alleviated alcoholic liver injury possibly by increasing the contents of bile acids that suppress the harmful bacteria.

Overall, alcohol intake caused intestinal microbiota disorder. The abundances of *Bacteroides*, *Blautia*, *Collinsella*, *Escherichia-Shigella*, *Enterococcus*, *[Ruminococcus]_gnavus_group*, *Prevotella*, *Romboutsia*, *Streptococcus*, *Bilophila*, and *Methylobacterium-Methylorubrum* were notably upregulated, while the abundance of Norank_f_Muribaculaceae was significantly reduced. These changes increased serum and hepatic lipid accumulation in mice and disrupted the intestinal barrier. C3G reversed the changes of microbiota and increased bile acids levels to alleviate the alcoholic liver injury (such as lipid accumulation), and gut mucosal barrier can also be partly restored. In summary, this study suggested that C3G has potentially beneficial effects on preventing alcohol-induced liver injury via modulating gut microbiota and metabolites.

## 5. Conclusions

The protective effect of C3G on alcohol-induced liver injury and its possible mechanism were studied from the perspective of gut microbiota and intestinal metabolites for the first time. Our results indicated that dietary supplementation of C3G could inhibit the accumulation of liver lipids and liver function abnormalities in the alcohol-fed mice, then improved alcohol-induced liver injury. Moreover C3G repaired the intestinal barrier destruction and ameliorated the structure of intestinal microbial disturbance in the alcohol-fed mice. The disturbance of intestinal microbial structure was characterized by a decrease in the number of bacteria that were unfavorable to the repair of liver function, meanwhile C3G increased bile acids levels in caecum. Taken together, Our work explains the molecular mechanism of C3G against alcoholic liver injury in terms of both microbiome and intestinal metabolomics. C3G attenuates alcohol-induced liver injury by modulating changes in the gut microbiota and metabolites, suggesting that it has the potential to develop functional foods that combat alcohol-induced liver injury; It can also promote the development and utilization of high value black rice.

## Data Availability

Raw data are available upon request.
